# Genetic Variants at the *NAT2* and *HLA‐DOA* are Associated With Anti‐Tuberculosis Drug‐Induced Liver Injury Susceptibility and Clinical Manifestations in Western Chinese Populations

**DOI:** 10.1002/iid3.70517

**Published:** 2026-09-15

**Authors:** Zhenzhen Zhao, Dongmei Wang, Yanjun Si, Yanhong Zhou, Hongxia Ruan, Binwu Ying, Hao Chen

**Affiliations:** ^1^ Department of Laboratory Medicine, West China Hospital Sichuan University Chengdu China; ^2^ Clinical Laboratory Medicine Research Center, West China Hospital Sichuan University Chengdu China; ^3^ Sichuan Clinical Research Center for Laboratory Medicine Chengdu China; ^4^ Institution of Medical and Engineering Integration for Molecular Diagnosis, West China Hospital Sichuan University Chengdu China; ^5^ Department of Science and Education Division Public Health Clinical Center of Chengdu Chengdu Sichuan China

**Keywords:** clinical manifestations, drug‐induced liver injury, HLA‐DOA, immunogenetic, tuberculosis

## Abstract

**Background:**

Anti‐tuberculosis drug‐induced liver injury (ATDILI) is one of the most prevalent and serious adverse reactions during anti‐tuberculosis treatment and can potentially lead to liver failure or mortality. This study aims to investigate whether genetic variants in the N‐acetyltransferase 2 gene (*NAT2*) and the HLA‐DOA gene (*HLA‐DOA)* are associated with ATDILI susceptibility and clinical manifestations in a Western Chinese population.

**Methods:**

A total of 1358 participants with active tuberculosis were enrolled and genotyped for four *NAT2* polymorphisms and five *HLA‐DOA* loci. Associations between candidate genetic variants and ATDILI were evaluated using logistic regression analyses, with multiple comparisons adjusted by Bonferroni correction.

**Results:**

The overall incidence of ATDILI was 28.4% (385/1358) in this cohort. Under a recessive model, *NAT2* rs1799930 was found to increase the risk of ATDILI (odds ratio [OR] = 1.88, 95% confidence interval [CI]: 1.20–2.95, *p* = 0.006), which remained significant after Bonferroni correction (adjusted *p* = 0.048). Meanwhile, while *HLA‐DOA* rs1367731 (OR = 0.41, 95% CI: 0.18–0.93, *p* = 0.033), rs6913008 (OR = 0.39, 95% CI: 0.17–0.89, *p* = 0.024), and rs9276975 (OR = 0.47, 95% CI: 0.23–0.98, *p* = 0.045) demonstrated a promising protective genomic characteristic that may mitigate the development of ATDILI. However, none of the reported *HLA‐DOA* associations remained statistically significant after applying Bonferroni corrections. Regarding clinical manifestations, *NAT2* rs1799930 and rs1799931 have been linked to poor ATDILI clinical presentations, whereas certain *HLA‐DOA* variants (rs1367731, rs6913008, and rs9276975) were possibly linked to milder ATDILI severity.

**Conclusion:**

Our findings preliminarily suggest that the *NAT2* and *HLA‐DOA* genetic variants may play a role in ATDILI susceptibility and clinical outcomes. *NAT2* rs1799930 represents a potential genetic risk marker, while *HLA‐DOA* variants may serve as protective factors warranting further validation. These findings may contribute to the precision management of ATDILI and the prevention of anti‐TB drug‐associated liver injury.

## 1 Introduction

In 2023, tuberculosis (TB) re‐emerged as the leading global cause of death from a single infectious agent, after having been surpassed by COVID‐19 over the preceding 3 years, with an estimated 10.8 million cases and 1.25 million deaths attributed to TB [1]. China has the third‐highest number of new TB cases, accounting for 6.8% of the global TB incidence [1]. Short‐course treatment strategies have been implemented to control this communicable disease, consisting of a 2‐month therapy with isoniazid (INH), rifampicin (RIF), ethambutol (EMB), and pyrazinamide (PZA), followed by a 4‐month treatment with INH and RIF [2]. However, anti‐TB combination medications could lead to adverse drug reactions, thereby complicating TB management. Anti‐tuberculosis drug‐induced liver injury (ATDILI) is the most prevalent and serious adverse reaction and can potentially lead to liver failure or mortality [3]. The incidence rates of ATDILI vary widely across different areas, ranging from 2% to 36% [4, 5, 6, 7]. ATDILI may result in treatment interruption, withdrawal, or regimen change. Furthermore, it also contributes to reduced treatment effectiveness, the emergence of drug resistance, non‐adherence, and treatment failure [8, 9]. Therefore, the assessment and monitoring of the anti‐TB drug‐induced hepatotoxicity remain a critical clinical priority for preventing liver injury.

N‐acetyltransferase 2 enzyme, encoded by the *NAT2* gene (*NAT2*), activates and deactivates arylamine and hydrazine drugs, including INH. Polymorphisms within this gene are responsible for the N‐acetylation polymorphism, which categorizes human populations into slow, intermediate, and rapid acetylator phenotypes [10]. Currently, a large body of evidence shows that *NAT2* genetic variants cause interindividual variability in the N‐acetylation of INH, where *NAT2* slow acetylator genotypes are more prone to result in ATDILI than fast acetylators [11, 12]. Azuma et al. have demonstrated that a *NAT2* genotype‐guided regimen significantly reduced the incidence of ATDILI, emphasizing the significance of the *NAT2* genotype assay in TB clinical practice [13]. Further, the Pharmacogenomics Knowledgebase (PharmGKB) database has indicated the utilization of *NAT2* genotype testing to optimize the daily dosage of INH [14]. However, the data regarding the association between *NAT2* genetic variants and ATDILI in Western China remains limited.

The pathogenesis of hepatotoxicity of anti‐TB drugs is complex and incompletely understood. The liver injury results not only from the direct toxic effects of anti‐TB agents on hepatocytes but also from the associated innate and adaptive immune response and inflammatory stress [15]. Metushi et al. detected anti‐INH autoantibodies in serum among patients with INH‐related liver failure [16]. Also, prior studies have shown that *human leukocyte antigen* (*HLA*) immunogenetic factors influence the susceptibility of TB patients to ATDILI. For example, a previous HLA class II typing study in an Indian population reported that the absence of HLA‐DQA1*01:02 and the presence of HLA‐DQB1*02:01 were relevant to the elevated risk of ATDILI [17]. Nicoletti et al. performed an HLA analysis in a European cohort and identified two significant signals associated with ATDILI occurrence risk, namely HLA‐C*12:02 and HLA‐B*52:01 [18]. However, there is a scarcity of data regarding the *HLA* risk factors related to ATDILI in the Western Chinese population.

*HLA‐DOA* belongs to the HLA class II alpha chain paralogues. HLA‐DOA forms a heterodimer with HLA‐DOB. The heterodimer, HLA‐DO, is an important modulator in the HLA class II‐restricted antigen presentation pathway through interaction with the HLA‐DM molecule in B cells and can modify the peptide exchange activity of HLA‐DM [19]. Recent studies have shown that the *HLA‐DOA* genetic variants could alter the susceptibility to multiple autoimmune diseases and infectious diseases, underscoring their importance in maintaining immune homeostasis [20, 21, 22]. Notably, the roles of the *HLA‐DOA* gene variants involved in liver disorders are gradually being deciphered; for example, rs2284191 has been identified as an independent *HLA‐DOA* genetic marker associated with susceptibility to hepatitis C virus (HCV) infection [21]. Unfortunately, research on the link between *HLA‐DOA* genetic markers and TB is scarce.

ATDILI is the leading cause of drug‐related hepatotoxicity. The purpose of the current study was to investigate whether genetic variants of *NAT2* and *HLA‐DOA* are associated with ATDILI susceptibility and clinical manifestations. Our study findings could promote a deeper understanding of the etiology of ATDILI and facilitate new approaches for preventing anti‐TB drug‐associated liver injury and optimizing TB management.

## 2 Patients and Methods

### 2.1 Ethics Statement

This study protocol was approved by the Ethical Committee of West China Hospital, Sichuan University (reference no. 829, 2019) as well as the Ethics Committee of the Public Health Clinical Center of Chengdu (reference no. YJ‐K2022‐06‐01). Informed consent was obtained from each participant, and this study was performed according to the Declaration of Helsinki principles.

### 2.2 Study Population

**Figure 1:**
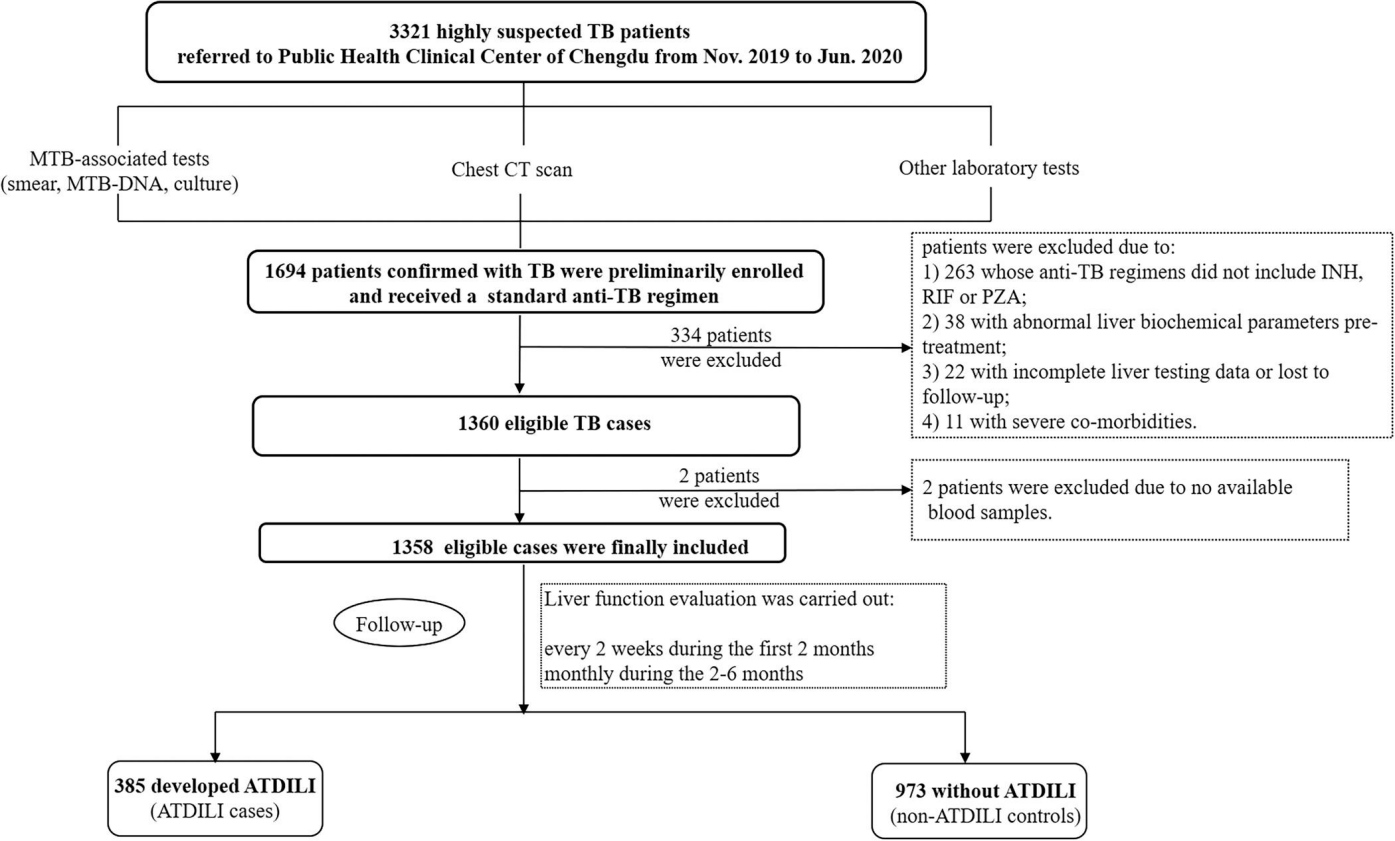
Study participants. ATDILI, anti‐tuberculosis drug‐induced liver injury; DILI, drug‐induced liver injury.

We consecutively recruited 3321 patients with presumptive TB referred to the Public Health Clinical Center of Chengdu from November 2019 to June 2020. In total, 1694 patients were confirmed with TB disease by professional respiratory physicians, according to typical TB symptoms, radiological evidence of *Mycobacterium tuberculosis* (MTB) infection, MTB‐associated etiological testing, and positive response to anti‐TB therapy. All patients included in our study were treated with a standard anti‐TB treatment regimen consisting of INH (300–400 mg/day), RIF (450–600 mg/day), PZA (1500–2000 mg/day), and EMB (750–900 mg/day) for the first 2 months, followed by INH, RIF, and EMB for 4 months. The specific exclusion criteria are listed in Figure 1: (1) patients with viral hepatitis, human immunodeficiency virus (HIV) infection, tumors, renal transplantation or other severe hepatic and extra‐hepatic co‐morbidities; (2) patients with aberrant liver function indicators before inclusion; (3) patients who lost to follow up or treatment interruption; (4) subjects who received non‐standard treatment regimen initially; (5) patients with incomplete electronic medical information; (6) unavailable blood samples from the participants. Ultimately, 1358 Western Chinese Han participants with a diagnosis of TB were enrolled in this study. Liver function tests were carried out every 2 weeks during the first 2 months and monthly afterward.

The cohort of the study was observed over a period of 6 months. According to whether they developed ATDILI, patients were divided into the ATDILI group and the anti‐TB treatment tolerance group (namely, the non‐ATDILI group). The definition of ATDILI and its severity have been described in detail previously [23]. When alanine aminotransferase (ALT) levels were ≥ 3× the upper limit of normal (ULN) together with hepatitis‐related symptoms, or ALT levels ≥ 5× the ULN with or without clinical manifestations, either situation was considered to be drug‐induced liver damage. All patients with ATDILI suspicion were reviewed by a panel of two physicians with expertise during the 6‐month follow‐up. The biochemical phenotypes of ATDILI were classified as hepatocellular (R ≥ 5), cholestatic (R ≤ 2), and mixed (2 < R < 5) patterns. The R was calculated as the value of serum [ALT/ALT ULN]/serum [alkaline phosphatase (ALP)/ALP ULN]. The principles of drug withdrawal are as follows: (1) When ALT < 3× ULN without obvious clinical symptoms or jaundice, hepatoprotective treatment can be administered under close observation, and some anti‐TB drugs with a high incidence of liver injury may be discontinued as appropriate. (2) When ALT ≥ 3× ULN, or total bilirubin (TBIL) ≥ 2× ULN: the anti‐TB drugs related to liver injury should be discontinued, hepatoprotective treatment should be given, close observation should be performed, and other potential factors causing liver injury should be eliminated. (3) When ALT ≥ 5× ULN, or ALT ≥ 3× ULN accompanied by symptoms such as jaundice, nausea, vomiting, and fatigue, or TBIL ≥ 3× ULN: all anti‐TB drugs should be discontinued immediately, and active hepatoprotective treatment should be carried out.

### 2.3 Candidate Polymorphisms Selection and Genotyping

Key genetic variants of the *NAT2* gene were selected, specifically focusing on four functionally significant single‐nucleotide polymorphisms (SNPs): rs1801279, rs1801280, rs1799930, and rs1799931. Utilizing Hein's established methodology, we systematically inferred the NAT2 acetylator phenotypes (slow, intermediate, and rapid) through comprehensive analysis of this four‐SNP panel [10]. Certain *HLA* alleles have been reported to affect patients' susceptibility to ATDILI, and candidate SNPs in the *HLA‐DOA* gene were selected by thoroughly searching the dbSNP database and the 1000 Genomes Project. Our rigorous selection criteria encompassed SNPs located in promoter regions, exonic sequences, and the 3′‐untranslated region (3′ UTR), with a stringent requirement of a minor allele frequency exceeding 0.05 among the Chinese Han Beijing. Finally, five *HLA‐DOA* SNPs (rs1367731, rs376892, rs6913008, rs9276975, and rs9276977) were included.

Peripheral blood was collected from each participant. Genomic DNA was extracted by employing QIAamp DNA Blood Mini Kits (Qiagen, Germany). Genotypes of the nine candidate SNPs analyzed in this study were detected by using a commercial 48‐Plex SNPscan Kit (Genesky Biotechnologies Inc., Shanghai, China). For quality control, approximately 10% of specimens were randomly selected and genotyped in duplicate, with 100% concordance.

### 2.4 Data Collection

The following parameters were collected for all enrolled patients. (1) demographic features: gender, age, drinking, and smoking status; (2) TB clinical characteristics: TB status, TB subtype, cough, low‐grade fever, hemoptysis, chest pain, night sweat, shortness of breath, weight loss and MTB smear result; (3) chest imaging findings: lung cavitation and pleural effusion; and (4) laboratory parameters at baseline and during ATDILI were described in detail in Supporting Information S1: Table S1.

### 2.5 Statistical Analysis

Categorical data were summarized using counts and percentages. Normality was assessed using the Shapiro–Wilk test. Normally distributed variables are presented as mean ± standard deviations (SDs), and non‐normally distributed variables as median (interquartile range [IQR]). Categorical variables were analyzed with the chi‐square test or Fisher's exact test, and continuous variables were compared using the Mann‐Whitney *U* test or Student's *t*‐test. Clinical features with a *p* value of less than 0.05 in the univariate analysis were included for multivariate logistic regression analysis to identify independent risk factors (at baseline) related to ATDILI. Associations between candidate SNPs and ATDILI were evaluated using unconditional logistic regression after adjusting for age and gender. In addition, the odds ratio (OR) with 95% confidence interval (CI) was used to assess the associations. Multiple comparisons were adjusted by Bonferroni correction. Statistical power was estimated using the genetic association study (GAS) power calculator (https://csg.sph.umich.edu/abecasis/gas_power_calculator/). An association analysis (*α* = 0.05) could detect a genotype relative risk of 1.50, and with 80% power in the overall (385 cases and 973 controls) analysis. The HaploView software was used to perform Haplotype analysis. The above‐described analyses were completed with SPSS 22.0 and PLINK 1.07. All statistical tests were two‐sided, and a *p* value of < 0.05 denoted statistical significance.

### 2.6 Functional Annotation

Polymorphisms rs376892, rs9276975, and rs9276977 were located in the *HLA‐DOA* gene 3′‐UTR. In this work, we evaluate the putative functions of these SNPs with the SNPinfo Web Server. In addition, we identified the *HLA* alleles that were well studied and found directly associated with drug‐induced liver injury (DILI), by searching for the PharmGKB database, the dbSNP database, and the PubMed database, and analyzed the tagging properties of *HLA‐DOA* alleles in relation to these certain *HLA* alleles. Further, we queried *HLA‐DOA* variants for the GTEx portal and obtained all expression quantitative trait loci (eQTL) of them.

## 3 Results

### 3.1 Patient Characteristics

The clinical characteristics and baseline quantitative laboratory data of the active TB patients are summarized in Tables 1 and 2, respectively. Ultimately, 1358 patients with TB were eligible for the study, 51.6% (701/1358) of whom were male. The mean age of the participants was 35.7 years. Significant differences in age and gender distribution were observed between patients with ATDILI and those without ATDILI. The ATDILI group was older and had more males than the non‐ATDILI group. The proportions of smokers and alcohol drinkers were 21.0% (81/385) and 16.4% (63/385) in the ATDILI cases, while 17.9% (174/973) and 12.6% (123/973) in the non‐ATDILI group, respectively. Higher percentages of smoking and alcohol consumption were observed in the ATDILI group, but these differences were not statistically significant. The majority (79.5%, 1079/1358) of the overall cohort exhibited pulmonary TB (PTB), while 8.8% (119/1358) had extra‐PTB (EPTB), and 11.7% (160/1358) had PTB combined with EPTB. Almost one‐quarter of patients with ATDILI had previously received anti‐TB treatment, which was significantly higher than in the non‐ATDILI group (23.4% vs. 11.6%, *p* = 0.001). The positive rates for fever and hemoptysis in the ATDILI group were 29.1% and 15.1%, respectively, which were considerably higher than those in the non‐ATDILI group (20.7% and 11.0%). The most common finding in chest imaging was lung cavitation (24.4%, 332/1358), followed by pleural effusion (22.9%, 311/1358). A significantly higher incidence of pleural effusion was observed in ATDILI patients compared with those without ATDILI (28.1% vs. 22.7%, *p* = 0.004).

**Table 1:** Demographic and clinical characteristics of TB patients with and without ATDILI.

Indexes	Overall (*n* = 1358)	ATDILI		*p* value^a^
		With (*n* = 385)	Without (*n* = 973)	
Age, mean ± SD (years)	35.7 ± 16.1	37.2 ± 16.2	35.1 ± 16.1	**0.032**
Gender, male (%)	701 (51.6)	258 (67.0)	443 (45.5)	**0.001**
Smoking, Yes/(Ever/No)	255 (18.8)	81 (21.0)	174 (17.9)	0.180
Drinking, Yes/(Ever/No)	186 (13.7)	63 (16.4)	123 (12.6)	0.072
TB status, *n* (%)				**0.001**
De novo	1155 (85.1)	295 (76.6)	860 (88.4)	
Retreated	203 (14.9)	90 (23.4)	113 (11.6)	
TB subtype, *n* (%)				0.352
PTB	1079 (79.5)	315 (81.6)	764 (78.5)	
EPTB	119 (8.8)	28 (7.3)	91 (9.4)	
PTB and EPTB	160 (11.7)	42 (10.9)	118 (12.1)	
General symptoms, *n* (%)				
Cough	928 (68.3)	268 (69.6)	660 (67.8)	0.525
Low‐grade fever	313 (23.0)	112 (29.1)	201 (20.7)	**0.001**
Hemoptysis	165 (12.2)	58 (15.1)	107 (11.0)	**0.048**
Chest pain	206 (15.2)	63 (16.4)	143 (14.7)	0.440
Night sweat	32 (2.4)	6 (1.6)	26 (2.7)	0.223
Shortness of breath	211 (15.5)	56 (14.5)	155 (15.9)	0.526
Weight loss	40 (2.9)	10 (2.6)	30 (3.1)	0.633
Chest image of TB, *n* (%)				
Cavitation	332 (24.4)	101 (26.2)	231 (23.7)	0.335
Pleural effusion	311 (22.9)	108 (28.1)	203 (22.7)	**0.004**
MTB positive smear, *n* (%)	220 (16.2)	52 (13.5)	168 (17.3)	0.138

*Note:* Bold values indicate statistical significance *p* < 0.05.

Abbreviations: ATDILI, anti‐tuberculosis drug‐induced liver injury; EPTB, extra‐pulmonary tuberculosis; MTB, *Mycobacterium tuberculosis*; PTB and EPTB, PTB combined with EPTB; PTB, pulmonary tuberculosis; TB, tuberculosis.

^a^ *p* values refer to comparisons between the ATDILI and non‐ATDILI groups.

**Table 2:** The baseline laboratory parameters of TB patients with and without ATDILI.

Parameters	Overall (*n* = 1358)	ATDILI		*p* value^a^
		With (*n* = 385)	Without (*n* = 973)	
TBIL (μmol/L)	7.1 (5.1–11.2)	6.8 (5.0–11.1)	7.3 (5.1–11.3)	0.291
DBIL (μmol/L)	3.2 (2.3–5.2)	3.1 (2.3–5.1)	3.1 (2.4–5.1)	0.304
IBIL (μmol/L)	3.9 (2.6–5.8)	3.8 (2.5–5.7)	4.0 (2.6–5.9)	0.292
ALT (U/L)	18.4 ± 7.8	21.1 ± 7.9	17.3 ± 7.6	**< 0.001**
AST (U/L)	22.0 ± 6.6	23.1 ± 8.2	21.6 ± 5.8	**< 0.001**
AST/ALT	1.3 ± 0.5	1.2 ± 0.5	1.4 ± 0.5	**< 0.001**
GGT (U/L)	22.0 (16.0–31.0)	22.0 (16.0–31.0)	21.0 (16.0–31.0)	0.489
ALP (U/L)	94.0 ± 48.3	95.1 ± 55.8	88.6 ± 45.0	**0.031**
Total protein (g/L)	73.8 ± 5.3	74.0 ± 5.0	73.8 ± 5.4	0.573
Albumin (g/L)	44.9 ± 4.2	44.7 ± 4.1	45.0 ± 4.2	0.235
Globulin (g/L)	28.9 ± 5.3	29.2 ± 5.4	28.7 ± 5.3	0.192
A/G	1.6 ± 0.4	1.6 ± 0.4	1.6 ± 0.4	0.146
CA (mmol/L)	2.4 ± 0.1	2.4 ± 0.1	2.4 ± 0.1	0.169
Glucose (mmol/L)	5.9 ± 2.0	5.9 ± 1.6	6.0 ± 2.1	0.426
UREA (mmol/L)	4.2 ± 1.6	4.2 ± 1.6	4.2 ± 1.6	0.522
CREA (μmol/L)	54.0 (46.0–64.0)	53.0 (44.0–64.0)	55.0 (46.0–64.8)	**0.028**
Cys‐c (mg/L)	0.9 ± 0.3	0.9 ± 0.2	0.9 ± 0.3	0.374
WBC (×10^9^/L)	6.0 ± 2.1	6.1 ± 2.0	5.9 ± 2.1	0.278
Neu (×10^9^/L)	4.0 ± 1.9	4.1 ± 1.9	3.9 ± 1.8	0.175
Lym (×10^9^/L)	1.5 ± 0.5	1.4 ± 0.5	1.5 ± 0.5	0.396
Mon (×10^9^/L)	0.4 ± 1.9	0.4 ± 0.2	0.4 ± 0.2	0.592
Eos (×10^9^/L)	0.1 (0.1–0.2)	0.1 (0.1–0.2)	0.1 (0.1–0.2)	0.310
RBC (×10^12^/L)	5.0 ± 2.6	5.0 ± 0.6	5.1 ± 3.0	0.718
Hb (g/L)	139.7 ± 19.3	139.6 ± 20.1	139.8 ± 19.0	0.884
Hct (%)	40.8 ± 6.3	40.9 ± 7.4	40.7 ± 5.9	0.690
PLT (×10^9^/L)	246.2 ± 89.8	252.2 ± 91.0	243.9 ± 89.2	0.121
PT (S)	13.7 ± 3.0	13.9 ± 3.9	13.6 ± 2.6	0.105
INR	1.1 ± 0.3	1.1 ± 0.3	1.1 ± 0.2	0.105
APTT (S)	34.5 ± 5.2	34.4 ± 5.1	34.6 ± 5.2	0.597
TT (S)	15.7 ± 1.6	15.7 ± 1.5	15.7 ± 1.7	0.716
ESR (mm/h)	25.0 (8.0–44.5)	25.0 (5.0–44.0)	26.0 (8.0–45.0)	0.058
CRP (mg/L)	1.8 (0.8–6.8)	2.3 (0.8–8.2)	1.6 (0.8–6.1)	**0.001**

*Note:* Normally distributed variables are presented as mean ± standard deviations (SD), and non‐normally distributed variables as median (interquartile range, IQR). Bold values indicate statistical significance *p* < 0.05.

Abbreviations: A/G, albumin/globulin; ALP, alkaline phosphatase; ALT, alanine aminotransferase; APTT, activated partial thromboplastin time; AST, aspartate aminotransferase; ATDILI, anti‐tuberculosis drug‐induced liver injury; CA, serum calcium; CREA, creatinine; CRP, C‐reactive protein; Cys‐c, Cystatin C; DBIL, direct bilirubin; Eos, eosinophils; ESR, erythrocyte sedimentation rate; GGT, gamma‐glutamyl transferase; Hb, hemoglobin; Hct, hematocrit; IBIL, indirect bilirubin; INR, international normalized ratio; Lym, lymphocytes; Mon, monocytes; Neu, neutrophils; PLT, platelet; PT, prothrombin time; RBC, red blood cell; TB, tuberculosis; TBIL, total bilirubin; TT, thrombin time; UREA, urea; WBC, white blood cell.

^a^ *p* values refer to comparisons between the ATDILI and non‐ATDILI groups.

Overall, most baseline laboratory parameters were comparable between the two groups of patients (Table 2). The patients with ATDILI had higher concentrations of ALT, aspartate aminotransferase (AST), ALP, and C‐reactive protein (CRP), but lower levels of AST/ALT ratio and creatinine (CREA) than individuals without ATDILI. In addition, the multivariate logistic regression analysis of baseline risk factors associated with ATDILI is summarized in Supporting Information S1: Table S2. The independent risk factors predictive of ATDILI included: male (OR = 2.35, 95% CI: 1.77–3.11), previous TB treatment (OR = 2.53, 95% CI: 1.78–3.60), low‐grade fever (OR = 1.52, 95% CI: 1.12–2.07), hemoptysis (OR = 1.55, 95% CI: 1.05–2.29), and baseline ALT ≥ 18.4 U/L (OR = 2.30, 95% CI: 1.69–3.12).

As shown in Table 3, 385 (28.4%) out of the 1358 patients treated with anti‐TB regimens were diagnosed with ATDILI, all of whom showed liver function abnormalities (i.e., ALT, AST, ALP, or TBIL > ULN). Regarding the biochemical phenotype of liver injury, 64.8% of patients suffered from hepatocellular injury, followed by mixed (30.0%, 110/385) and cholestatic (5.2%, 19/385) damage.

**Table 3:** Characteristics of patients suffered from ATDILI.

Features	ATDILI (*n* = 385)	
	Peak	Baseline
Biochemical phenotype of liver injury		
Hepatocellular, *n* (%)	238 (64.8)	NA
Cholestatic, *n* (%)	19 (5.2)	NA
Mixed, *n* (%)	110 (30.0)	NA
Laboratory tests profiles		
ALT (U/L)	170.5 (131.0–259.5)	20.0 (15.0–26.0)
AST (U/L)	124.0 (84.0–217.0)	23.0 (18.0–28.0)
Total protein (g/L)	70.5 ± 8.2	74 ± 5.0
Albumin (g/L)	41.8 ± 6.7	44.7 ± 4.1
TBIL (μmol/L)	10.9 (7.2–16.7)	6.8 (5.0–11.1)
DBIL (μmol/L)	4.8 (3.2–8.3)	3.1 (2.3–5.1)
ALP (U/L)	116.7 ± 111.3	95.1 ± 55.8
GGT (U/L)	70.0 (40.0–121.0)	22.0 (16.0–31.0)
INR	1.1 ± 0.4	1.1 ± 0.3
WBC (10^9^/L)	5.8 ± 2.4	6.1 ± 2.0
RBC (10^12^/L)	4.9 ± 0.7	5.0 ± 0.6
Hb (g/L)	141.6 ± 21.8	139.6 ± 20.1
PLT (10^9^/L)	209.3 ± 81.7	252.2 ± 91.0

*Note:* Normally distributed variables are presented as mean ± standard deviations (SD), and non‐normally distributed variables as median (interquartile range, IQR).

Abbreviations: ALP, alkaline phosphatase; ALT, alanine aminotransferase; AST, aspartate aminotransferase; ATDILI, anti‐tuberculosis drug‐induced liver injury; DBIL, direct bilirubin; GGT, gamma‐glutamyl transferase; Hb, hemoglobin; INR, international normalized ratio; NA, not available; PLT, platelet; RBC, red blood cell; TBIL, total bilirubin; WBC, white blood cell.

### 3.2 Associations and Effects of Candidate Variants on ATDILI

Candidate genetic variants and their characteristics are summarized in Supporting Information S1: Table S3. *NAT2* locus rs1801279 frequency in our sample is consistent with previous studies, which reported a G allele frequency of 100% in Asian. No deviations from the Hardy–Weinberg equilibrium were observed for the other eight SNPs across the TB patients (all *p* > 0.05). The allele, genotype, and inherited model frequency distributions of the gene polymorphisms analyzed are listed in Table 4.

**Table 4:** SNPs associated with ATDILI risk.

SNP	Allele (M/m)	Analyze model		ATDILI patients (*n* = 385)	Non‐ATDILI controls (*n* = 973)	*p* value	OR (95% CI)	*p* value^a^
*NAT2*								
rs1801280	T/C	Genotype	CC	1 (0.3)	0	NA	NA	
			TC	36 (9.4)	73 (7.5)	0.253	1.28 (0.84–1.94)	
			TT	348 (90.3)	900 (92.5)	Reference		
		Allele	C	38 (4.9)	73 (3.8)	0.252	1.28 (0.84–1.93)	
			T	732 (95.1)	1873 (96.2)	Reference		
		Dominant model	CC + TC	37 (9.6)	73 (7.5)	0.305	1.25 (0.82–1.90)	
			TT	348 (90.4)	900 (92.5)	Reference		
		Recessive model	CC	1 (0.3)	0	NA	NA	
			TC + TT	384 (99.7)	973 (100.0)	Reference		
rs1799930	G/A	Genotype	AA	38 (9.9)	49 (5.0)	**0.001**	2.19 (1.40–3.45)	**0.008**
			GA	149 (38.7)	364 (37.4)	0.251	1.16 (0.90–1.49)	
			GG	198 (51.4)	560 (57.6)	Reference		
		Allele	A	225 (29.2)	462 (23.7)	**0.010**	1.29 (1.06–1.56)	0.079
			G	545 (70.8)	1484 (76.3)	Reference		
		Dominant model	AA + GA	187 (48.6)	413 (42.4)	0.068	1.25 (0.98–1.60)	
			GG	198 (51.4)	560 (57.6)	Reference		
		Recessive model	AA	38 (9.9)	49 (5.0)	**0.006**	1.88 (1.20–2.95)	**0.048**
			GA + GG	347 (90.1)	924 (95.0)	Reference		
rs1799931	G/A	Genotype	AA	8 (2.1)	20 (2.1)	0.995	1.00 (0.43–2.29)	
			GA	101 (26.2)	265 (27.2)	0.708	0.95 (0.73–1.24)	
			GG	276 (71.7)	688 (70.7)	Reference		
		Allele	A	117 (15.2)	305 (15.7)	0.794	0.97 (0.76–1.23)	
			G	653 (84.8)	1641 (84.3)	Reference		
		Dominant model	AA + GA	109 (28.3)	285 (29.3)	0.779	0.96 (0.74–1.26)	
			GG	276 (71.7)	688 (70.7)	Reference		
		Recessive model	AA	8 (2.1)	20 (2.1)	0.971	0.98 (0.42–2.30)	
			GA + GG	377 (97.9)	953 (97.9)	Reference		
*HLA‐DOA*								
rs1367731	C/T	Genotype	TT	7 (1.8)	40 (4.1)	**0.039**	0.43 (0.20–0.98)	0.312
			TC	128 (33.3)	315 (32.4)	0.972	1.00 (0.78–1.29)	
			CC	250 (64.9)	618 (63.5)	Reference		
		Allele	T	142 (18.4)	395 (20.3)	0.295	0.89 (0.71–1.11)	
			C	628 (81.6)	1551 (79.7)	Reference		
		Dominant model	TT + TC	135 (35.1)	355 (36.5)	0.709	0.95 (0.74–1.23)	
			CC	250 (64.9)	618 (63.5)	Reference		
		Recessive model	TT	7 (1.8)	40 (4.1)	**0.033**	0.41 (0.18–0.93)	0.267
			TC + CC	378 (98.2)	933 (95.9)	Reference		
rs376892	G/A	Genotype	AA	20 (5.2)	64 (6.6)	0.266	0.74 (0.44–1.26)	
			GA	149 (38.7)	396 (40.7)	0.371	0.89 (0.70–1.14)	
			GG	216 (56.1)	513 (52.7)	Reference		
		Allele	A	189 (24.6)	524 (26.9)	0.264	0.89 (0.73–1.09)	
			G	581 (75.4)	1422 (73.1)	Reference		
		Dominant model	AA + GA	169 (43.9)	460 (47.3)	0.346	0.89 (0.70–1.13)	
			GG	216 (56.1)	513 (52.7)	Reference		
		Recessive model	AA	20 (5.2)	64 (6.6)	0.378	0.79 (0.47–1.34)	
			GA + GG	365 (94.8)	909 (93.4)	Reference		
rs6913008	C/T	Genotype	TT	7 (1.8)	42 (4.3)	**0.027**	0.41 (0.18–0.93)	0.216
			TC	128 (33.2)	315 (32.4)	0.992	1.00 (0.78–1.29)	
			CC	250 (65.0)	616 (63.3)	Reference		
		Allele	T	142 (18.4)	399 (20.5)	0.227	0.87 (0.70–1.09)	
			C	628 (81.6)	1547 (79.5)	Reference		
		Dominant model	TT + TC	135 (35.1)	357 (36.7)	0.617	0.94 (0.73–1.21)	
			CC	250 (64.9)	616 (63.3)	Reference		
		Recessive model	TT	7 (1.8)	42 (4.3)	**0.024**	0.39 (0.17–0.89)	0.196
			TC + CC	378 (98.2)	931 (95.7)	Reference		
rs9276975	C/T	Genotype	TT	9 (2.3)	45 (4.6)	0.051	0.49 (0.24–1.02)	
			TC	134 (34.8)	336 (34.5)	0.846	0.98 (0.76–1.25)	
			CC	242 (62.9)	592 (60.9)	Reference		
		Allele	T	152 (19.7)	426 (21.9)	0.256	0.88 (0.71–1.09)	
			C	618 (80.3)	1520 (78.1)	Reference		
		Dominant model	TT + TC	143 (37.1)	381 (39.2)	0.613	0.94 (0.73–1.20)	
			CC	242 (62.9)	592 (60.8)	Reference		
		Recessive model	TT	9 (2.3)	45 (4.6)	**0.045**	0.47 (0.23–0.98)	0.361
			TC + CC	376 (97.7)	928 (95.4)	Reference		
rs9276977	G/A	Genotype	AA	11 (2.9)	45 (4.6)	0.140	0.60 (0.31–1.19)	
			GA	134 (34.8)	335 (34.4)	0.927	0.99 (0.77–1.27)	
			GG	240 (62.3)	593 (60.9)	Reference		
		Allele	A	156 (20.3)	425 (21.8)	0.354	0.90 (0.73–1.12)	
			G	614 (79.7)	1521 (78.2)	Reference		
		Dominant model	AA + GA	145 (37.7)	380 (39.1)	0.668	0.95 (0.74–1.21)	
			GG	240 (62.3)	593 (60.9)	Reference		
		Recessive model	AA	11 (2.9)	45 (4.6)	0.111	0.58 (0.29–1.14)	
			GA + GG	374 (97.1)	928 (95.4)	Reference		

*Note: p* value has been adjusted for age and gender. Bold values indicate statistical significance *p* < 0.05.

Abbreviations: ATDILI, anti‐tuberculosis drug‐induced liver injury; NA, not available; SNP, single nucleotide polymorphism.

^a^ *p* values were adjusted for multiple comparisons using the Bonferroni correction.

*NAT2* variants were associated with ATDILI predisposition. For rs1799930, the A allele carriers showed an elevated risk of developing ATDILI compared to those carrying the G allele (OR = 1.29, 95% CI: 1.06–1.56, *p* = 0.010, Table 4), and AA genotype frequency was significantly higher in the ATDILI cases than in the non‐ATDILI controls (AA vs. GG, OR = 2.19, 95% CI: 1.40–3.45, *p* = 0.001, Bonferroni *p* = 0.008). In addition, patients with the AA genotype of rs1799930 were 1.88‐fold more likely to develop ATDILI than those with a G allele‐containing genotype (AA vs. GA + GG, *p* = 0.006, Bonferroni‐adjusted *p* = 0.048). No statistically significant difference was found between the two groups in the different genotypes of other SNPs (all *p* > 0.05). *NAT2* acetylation status was further explored with the 4‐SNP panel. The distribution of the rapid, intermediate, and slow acetylator *NAT2* genotypes in cases and controls is shown in Supporting Information S1: Table S4A. Expectedly, the *NAT2* slow acetylator phenotype was a strong marker for identifying individuals at elevated risk of ATDILI (OR = 1.84, 95% CI: 1.39–2.44, *p* < 0.001, Supporting Information S1: Table S4B).

The relationship between *HLA‐DOA* variations and ATDILI was investigated for the first time in this study. We found the following variants that might reduce the odds of ATDILI occurrence during the anti‐TB treatment: rs1367731, rs6913008, and rs9276975. Specifically, only 7 of the 385 identified cases of ATDILI exhibited a homozygous TT mutation in the rs1367731 locus, which was associated with a decreased risk of ATDILI under both the genotype and recessive models (OR = 0.43, 95% CI = 0.20–0.98, *p* = 0.039 and OR = 0.41, 95% CI: 0.18–0.93, *p* = 0.033, respectively, Table 4). Similar results were observed in SNP rs6913008, where TT genotype carriers were significantly less likely to develop ATDILI (TT vs. CC, OR = 0.41, 95% CI: 0.18–0.93, *p* = 0.027; TT vs. TC + CC, OR = 0.39, 95% CI: 0.17–0.89, *p* = 0.024, Table 4). Additionally, TB patients with the TT genotype in rs9276975 exhibited a marginally lower risk of ATDILI compared to those with TC or CC genotypes (TT vs. TC + CC, OR = 0.47, 95% CI: 0.23–0.98, *p* = 0.045). However, it should be noted that all associations reported were not statistically significant after Bonferroni correction. Utilizing linkage disequilibrium (LD) information from the 1000 Genomes Project, the three SNPs exhibiting near‐significance had high LD with each other (*r* ^2^ = 0.90), thereby indicating that rs9276975, rs6913008, and rs1367731 could be considered as proxies for one another. The rs9276975–rs6913008–rs1367731 haplotypes were determined in our analysis. Disappointingly, the SNPs combination did not show enhanced protective effect against ATDILI risk, and no associations were found between the *HLA‐DOA* haplotypes and susceptibility to ATDILI (*p* > 0.05 for all), as demonstrated in Supporting Information S1: Table S5.

### 3.3 Implications of *NAT2* and *HLA‐DOA* Variants on ATDILI Clinical Characteristics

**Figure 2:**
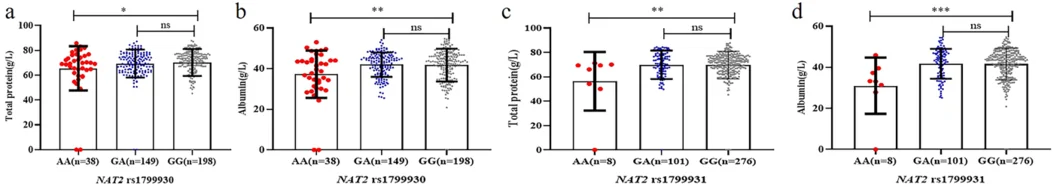
Association of *NAT2* SNPs with clinical indices during ATDILI occurrence in patients. (a) and (b) The association between rs1799930 genotypes with the abundance of TP and ALB in the serum. (c) and (d) The link between rs1799931 genotypes with the abundance of serum TP and ALB. Data shown are mean ± SD. *p < 0.05; **p < 0.01; ***p < 0.001; ns, not significant. ALB, albumin; TP, total protein.

**Figure 3:**
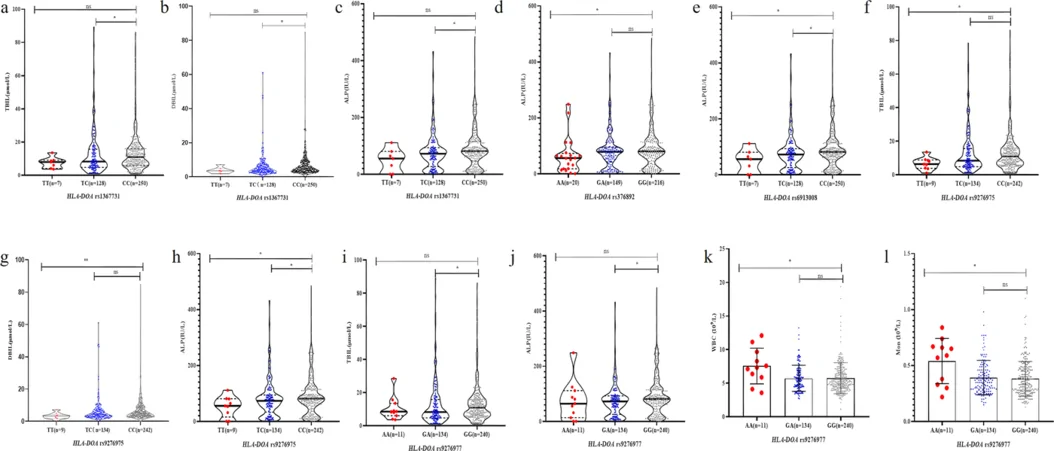
Association of *HLA‐DOA* SNPs with clinical indices during ATDILI occurrence in patients. (a–j) The association between genotypes rs1367731, rs376892, rs6913008, rs9276975, and rs9276977 and the serum levels of TBIL, DBIL, and ALP. Data shown are median (IQR). (k) and (l) The association of rs9276977 genotypes with the abundance of WBC and Mon in the peripheral circulation. Data shown are mean ± SD. *p < 0.05; **p < 0.01; ***p < 0.001; ns, not significant. ALP, alkaline phosphatase; DBIL, direct bilirubin; Mon, monocytes; TBIL, total bilirubin; WBC, white blood cell.

The potential functions of the *NAT2* and *HLA‐DOA* variants, as well as the association with ATDILI patients' clinical manifestations, require further investigation. Therefore, a thorough investigation was performed to elucidate the relationships between *NAT2* and *HLA‐DOA* gene polymorphisms and clinical characteristics of ATDILI patients. Tables 5 and 6, Supporting Information S1: Tables S6 and S7, and Figures 2 and 3 provide a comprehensive overview. This analysis may provide significant additional value in patients with new gene variants.

**Table 5:** Association between *NAT2* SNPs and laboratory characteristics at ATDILI diagnosis.

SNP	Peak parameters	ATDILI patients (*n* = 385)			*p* value^a^	Bonferroni *p* value^a^	*p* value^b^	Bonferroni *p* value^b^
		Genotype						
rs1801280		CC (*n* = 1)	TC (*n* = 36)	TT (*n* = 348)				
	ESR (mm/h)	0.0	24.0 (4.0–56.0)	23.0 (3.0–41.0)	NA		0.628	
	CRP (mg/L)	4.2	4.9 (1.2–19.9)	4.0 (1.3–11.0)	NA		0.570	
	WBC (10^9^/L)	5.6	5.9 ± 2.8	5.8 ± 2.4	NA		0.767	
	Neu (10^9^/L)	3.2	4.0 ± 2.7	3.7 ± 2.1	NA		0.531	
	Lym (10^9^/L)	1.7	1.3 ± 0.8	1.5 ± 0.7	NA		0.285	
	Mon (10^9^/L)	0.5	0.4 ± 0.2	0.4 ± 0.2	NA		0.465	
	TBIL (μmol/L)	29.0	11.0 (7.6–16.0)	10.8 (6.9–16.8)	NA		0.804	
	DBIL (μmol/L)	18.9	4.7 (3.6–7.5)	4.6 (3.1–8.3)	NA		0.649	
	ALT (U/L)	315.0	152.0 (116.3–208.5)	172.0 (133.3–260.8)	NA		0.050	
	AST (U/L)	469.0	106.5 (79.3–225.3)	125.0 (84.0–217.0)	NA		0.556	
	GGT (U/L)	759.0	64.5 (43.3–111.8)	69.0 (36.0–120.8)	NA		0.846	
	ALP (U/L)	248.0	86.5 (67.3–127.8)	90.0 (68.0–116.0)	NA		0.744	
	Total protein (g/L)	80.0	66.0 ± 7.9	69.8 ± 12.2	NA		0.071	
	Albumin (g/L)	48.4	39.3 ± 6.9	41.6 ± 8.0	NA		0.105	
	Globulin (g/L)	31.6	27.0 ± 4.8	28.4 ± 7.0	NA		0.215	
rs1799930		AA (*n* = 38)	GA (*n* = 149)	GG (*n* = 198)				
	ESR (mm/h)	24.0 (3.0–38.5)	20.0 (3.0–39.0)	24.0 (4.0–44.0)	0.948		0.216	
	CRP (mg/L)	4.4 (1.2–23.6)	4.6 (1.7–14.5)	4.0 (1.1–10.0)	0.670		0.161	
	WBC (10^9^/L)	5.5 ± 1.9	6.0 ± 2.8	5.7 ± 2.1	0.713		0.205	
	Neu (10^9^/L)	3.6 ± 1.9	4.0 ± 2.6	3.6 ± 1.8	> 0.999		0.147	
	Lym (10^9^/L)	1.3 ± 0.5	1.4 ± 0.6	1.5 ± 0.8	0.408		0.816	
	Mon (10^9^/L)	0.4 ± 0.2	0.4 ± 0.2	0.4 ± 0.2	0.541		0.728	
	TBIL (μmol/L)	10.7 (6.7–17.6)	10.5 (7.0–17.6)	11.0 (7.2–16.0)	0.850		0.718	
	DBIL (μmol/L)	4.1 (2.9–5.9)	4.9 (3.0–8.9)	4.6 (3.3–7.8)	0.215		0.837	
	ALT (U/L)	176.0 (142.3–243.0)	168.0 (131.0–259.0)	175.0 (130.0–264.3)	0.977		0.682	
	AST (U/L)	148.0 (78.8–222.5)	130.0 (85.0–213.0)	120.0 (80.5–234.0)	0.718		0.464	
	GGT (U/L)	74.0 (36.8–130.3)	62.0 (33.0–117.5)	71.0 (39.3–120.0)	0.905		0.336	
	ALP (U/L)	92.0 (71.5–121.5)	83.0 (67.0–115.0)	91.5 (68.8–121.3)	0.861		0.155	
	Total protein (g/L)	65.5 ± 17.9	69.4 ± 11.2	70.2 ± 10.9	**0.032**	0.256	0.467	
	Albumin (g/L)	37.3 ± 11.6	42.00 ± 6.0	41.8 ± 8.1	**0.004**	**0.032**	0.777	
	Globulin (g/L)	28.6 ± 10.8	27.9 ± 6.1	28.5 ± 6.4	0.945		0.376	
rs1799931		AA (*n* = 8)	GA (*n* = 101)	GG (*n* = 276)				
	ESR (mm/h)	31.5 (3.0–58.5)	23.0 (4.0–44.0)	22.0 (3.0–40.0)	0.726		0.454	
	CRP (mg/L)	12.2 (2.2–24.3)	5.7 (1.9–14.4)	3.2 (1.2–9.8)	0.278		0.058	
	WBC (10^9^/L)	5.9 ± 2.1	6.2 ± 2.4	5.7 ± 2.4	0.813		0.077	
	Neu (10^9^/L)	3.8 ± 2.0	4.0 ± 2.1	3.7 ± 2.2	0.895		0.155	
	Lym (10^9^/L)	1.5 ± 0.5	1.5 ± 1.0	1.4 ± 0.5	0.645		0.302	
	Mon (10^9^/L)	0.3 ± 0.1	0.4 ± 0.2	0.4 ± 0.2	0.436		0.064	
	TBIL (μmol/L)	6.8 (4.1–11.9)	10.7 (7.4–15.7)	10.9 (6.8–17.2)	0.097		0.761	
	DBIL (μmol/L)	3.5 (2.3–6.1)	5.1 (3.5–7.4)	4.5 (3.0–8.4)	0.262		0.584	
	ALT (U/L)	155.0 (146.3–195.8)	178.0 (131.0–289.5)	169.0 (130.0–256.0)	0.733		0.445	
	AST (U/L)	117.0 (97.5–134.3)	124.5 (86.0–249.8)	122.5 (79.3–216.5)	0.955		0.583	
	GGT (U/L)	72.0 (50.3–128.5)	65.0 (36.0–120.0)	69.0 (36.3–120.8)	0.765		0.950	
	ALP (U/L)	85.0 (44.3–100.0)	87.0 (60.0–117.0)	90.0 (72.0–118.8)	0.334		0.161	
	Total protein (g/L)	56.3 ± 24.1	69.9 ± 11.7	69.6 ± 11.4	**0.002**	**0.016**	0.866	
	Albumin (g/L)	31.1 ± 13.7	41.7 ± 7.3	41.6 ± 7.7	**< 0.001**	**0.002**	0.868	
	Globulin (g/L)	25.8 ± 12.5	28.5 ± 7.1	28.3 ± 6.5	0.302		0.814	

*Note:* Normally distributed variables are presented as mean ± standard deviations (SD), and non‐normally distributed variables as median (interquartile range, IQR). Bold values indicate statistical significance p < 0.05.

Abbreviations: ALP, alkaline phosphatase; ALT, alanine aminotransferase; AST, aspartate aminotransferase; ATDILI, anti‐tuberculosis drug‐induced liver injury; CRP, C‐reactive protein; DBIL, direct bilirubin; ESR, erythrocyte sedimentation rate; GGT, gamma‐glutamyl transferase; Lym, lymphocytes; Mon, monocytes; NA, not available; Neu, neutrophils; SNP, single nucleotide polymorphism; TBIL, total bilirubin; WBC, white blood cell.

^a^ *p* value for the comparison between the mm (homozygous minor allele) genotype and the MM (homozygous major allele) genotype.

^b^ *p* value for the comparison between the mM (heterozygous) genotype and the MM genotype. Multiple comparisons were adjusted using the Bonferroni correction.

**Table 6:** Association between *HLA‐DOA* SNPs and laboratory characteristics at ATDILI diagnosis.

SNP	Peak parameters	ATDILI patients (*n* = 385)			*p* value^a^	Bonferroni *p* value^a^	*p* value^b^	Bonferroni *p* value^b^
		Genotype						
rs1367731		TT (*n* = 7)	TC (*n* = 128)	CC (*n* = 250)				
	ESR (mm/h)	17.0 (3.0–38.0)	24.0 (4.0–43.0)	23.0 (4.0–40.0)	0.631		0.588	
	CRP (mg/L)	3.9 (0.9–13.4)	3.9 (1.7–14.1)	3.9 (1.4–8.7)	0.838		0.641	
	WBC (10^9^/L)	7.2 ± 2.6	5.8 ± 2.0	5.8 ± 2.5	0.144		0.967	
	Neu (10^9^/L)	4.1 ± 1.6	3.7 ± 1.9	3.8 ± 2.2	0.719		0.703	
	Lym (10^9^/L)	1.4 ± 0.7	1.6 ± 0.7	1.5 ± 0.7	0.900		0.239	
	Mon (10^9^/L)	0.5 ± 0.2	0.4 ± 0.2	0.4 ± 0.2	0.293		0.993	
	TBIL (μmol/L)	8.0 (4.1–9.1)	8.3 (4.8–15.7)	11.1 (6.2–16.4)	0.122		**0.033**	0.264
	DBIL (μmol/L)	3.5 (2.1–4.9)	4.3 (2.8–6.9)	4.9 (3.2–8.8)	0.072		**0.035**	0.280
	ALT (U/L)	155.0 (144.0–368.0)	147.0 (113.3–220.5)	156.0 (122.0–243.5)	0.711		0.253	
	AST (U/L)	87.0 (76.0–142.0)	97.0 (57.0–214.0)	97.0 (65.0–191.5)	0.740		0.611	
	GGT (U/L)	37.0 (21.0–74.0)	56.0 (27.0–113.0)	59.0 (32.0–95.8)	0.182		0.569	
	ALP (U/L)	56.0 (1.0–81.0)	74.0 (31.3–95.0)	82.0 (47.8–116.0)	0.052		**0.039**	0.312
	Total protein (g/L)	56.8 ± 23.1	67.0 ± 18.1	63.2 ± 21.6	0.443		0.092	
	Albumin (g/L)	40.2 ± 8.1	39.5 ± 11.0	38.6 ± 12.7	0.741		0.517	
	Globulin (g/L)	19.6 ± 11.9	27.0 ± 9.5	25.1 ± 10.2	0.156		0.091	
rs376892		AA (*n* = 20)	GA (*n* = 149)	GG (*n* = 216)				
	ESR (mm/h)	25.0 (3.0–41.0)	23.0 (4.0–42.0)	23.0 (4.0–39.5)	0.880		0.916	
	CRP (mg/L)	5.9 (2.4–19.5)	3.2 (1.4–9.1)	4.4 (1.5–8.7)	0.389		0.476	
	WBC (10^9^/L)	6.6 ± 2.3	5.8 ± 2.1	5.8 ± 2.6	0.220		0.889	
	Neu (10^9^/L)	4.6 ± 2.2	3.6 ± 1.7	3.8 ± 2.3	0.127		0.273	
	Lym (10^9^/L)	1.4 ± 0.7	1.5 ± 0.6	1.5 ± 0.8	0.804		0.576	
	Mon (10^9^/L)	0.5 ± 0.2	0.4 ± 0.2	0.4 ± 0.2	0.225		0.676	
	TBIL (μmol/L)	9.2 (6.6–13.5)	8.9 (5.6–17.6)	9.5 (5.8–15.7)	0.913		0.722	
	DBIL (μmol/L)	5.2 (2.7–8.1)	4.5 (3.2–7.8)	4.7 (3.1–8.3)	0.975		0.982	
	ALT (U/L)	141.0 (54.0–180.5)	164.0 (123.3–245.5)	149.0 (117.0–229.5)	0.413		0.081	
	AST (U/L)	96.5 (39.5–199.5)	114.0 (63.0–232.0)	92.5 (64.0–188.5)	0.659		0.563	
	GGT (U/L)	58.5 (30.8–80.0)	57.0 (27.8–118.0)	59.0 (32.0–93.0)	0.682		0.710	
	ALP (U/L)	56.5 (18.3–73.5)	79.0 (43.8–96.0)	81.0 (46.0–116.0)	**0.022**	0.176	0.340	
	Total protein (g/L)	67.4 ± 16.3	66.4 ± 18.6	62.7 ± 22.0	0.351		> 0.999	
	Albumin (g/L)	42.0 ± 6.1	39.2 ± 11.6	38.4 ± 12.8	0.222		0.578	
	Globulin (g/L)	29.1 ± 10.1	26.8 ± 9.3	24.6 ± 10.3	0.060		**0.039**	0.312
rs6913008		TT (*n* = 7)	TC (*n* = 128)	CC (*n* = 250)				
	ESR (mm/h)	17.0 (3.0–38.0)	23.5 (4.0–43.5)	23.0 (4.0–40.0)	0.624		0.643	
	CRP (mg/L)	3.9 (0.9–13.4)	3.8 (1.7–14.0)	3.9 (1.4–8.8)	0.831		0.661	
	WBC (10^9^/L)	7.2 ± 2.6	5.9 ± 2.1	5.8 ± 2.5	0.136		0.809	
	Neu (10^9^/L)	4.1 ± 1.6	3.7 ± 1.9	3.7 ± 2.2	0.697		0.930	
	Lym (10^9^/L)	1.4 ± 0.7	1.6 ± 0.7	1.5 ± 0.7	0.898		0.251	
	Mon (10^9^/L)	0.5 ± 0.2	0.4 ± 0.2	0.4 ± 0.2	0.282		0.822	
	TBIL (μmol/L)	8.0 (4.1–9.1)	8.3 (4.9–15.7)	11.1 (6.1–16.4)	0.131		0.072	
	DBIL (μmol/L)	3.5 (2.1–4.9)	4.3 (2.8–7.1)	4.9 (3.2–8.8)	0.073		0.049	
	ALT (U/L)	155.0 (144.0–268.0)	147.0 (113.3–220.5)	156.0 (122.0–243.5)	0.711		0.254	
	AST (U/L)	87.0 (76.0–142.0)	96.0 (57.0–214.0)	98.0 (65.0–191.5)	0.737		0.574	
	GGT (U/L)	37.0 (21.0–74.0)	56.0 (27.0–113.0)	59.0 (32.0–95.8)	0.182		0.567	
	ALP (U/L)	56.0 (1.0–81.0)	73.0 (26.0–95.0)	82.0 (53.3–116.0)	**0.049**	0.392	**0.017**	0.136
	Total protein (g/L)	56.8 ± 23.1	67.0 ± 18.1	63.2 ± 21.6	0.444		0.089	
	Albumin (g/L)	40.2 ± 8.1	39.4 ± 11.0	38.6 ± 12.7	0.743		0.537	
	Globulin (g/L)	19.6 ± 11.9	27.0 ± 9.5	25.1 ± 10.1	0.157		0.078	
rs9276975		TT (*n* = 9)	TC (*n* = 134)	CC (*n* = 242)				
	ESR (mm/h)	22.0 (3.5–40.0)	23.0 (3.8–41.0)	24.0 (4.0–41.0)	0.817		0.746	
	CRP (mg/L)	3.9 (1.4–20.5)	3.3 (1.4–9.0)	4.0 (1.5–9.3)	0.868		0.668	
	WBC (10^9^/L)	6.9 ± 2.4	5.8 ± 2.1	5.8 ± 2.5	0.222		0.952	
	Neu (10^9^/L)	3.9 ± 1.4	3.6 ± 1.9	3.8 ± 2.2	0.888		0.497	
	Lym (10^9^/L)	1.5 ± 0.7	1.6 ± 0.7	1.5 ± 0.7	0.965		0.224	
	Mon (10^9^/L)	0.5 ± 0.2	0.4 ± 0.2	0.4 ± 0.2	0.309		0.655	
	TBIL (μmol/L)	6.4 (3.9–8.9)	8.6 (4.9–15.9)	11.1 (6.1–16.4)	**0.031**	0.248	0.098	
	DBIL (μmol/L)	3.3 (1.9–4.2)	4.4 (3.0–7.2)	4.9 (3.3–8.8)	**0.009**	0.072	0.084	
	ALT (U/L)	155.0 (146.5–280.0)	146.5 (113.0–221.0)	156.0 (122.5–242.5)	0.520		0.239	
	AST (U/L)	81.0 (42.5–130.5)	99.0 (57.5–198.5)	97.0 (65.5–192.0)	0.282		0.619	
	GGT (U/L)	37.0 (23.5–74.0)	58.0 (27.0–112.0)	59.0 (31.5–96.5)	0.229		0.729	
	ALP (U/L)	56.0 (16.5–81.5)	75.0 (34.0–95.0)	82.0 (48.0–116.0)	**0.048**	0.384	**0.050**	0.400
	Total protein (g/L)	61.5 ± 22.1	67.0 ± 17.7	63.0 ± 21.8	0.841		0.070	
	Albumin (g/L)	40.9 ± 7.7	39.5 ± 11.4	38.5 ± 12.6	0.578		0.455	
	Globulin (g/L)	22.9 ± 12.3	27.0 ± 9.1	25.0 ± 10.3	0.548		0.077	
rs9276977		AA (*n* = 11)	GA (*n* = 134)	GG (*n* = 240)				
	ESR (mm/h)	23.0 (3.8–39.8)	24.0 (4.0–41.0)	23.0 (4.0–41.0)	0.857		0.691	
	CRP (mg/L)	3.9 (1.4–40.8)	3.5 (1.4–9.6)	3.9 (1.4–8.9)	0.601		0.855	
	WBC (10^9^/L)	7.6 ± 2.7	5.7 ± 1.9	5.8 ± 2.6	**0.029**	0.232	0.707	
	Neu (10^9^/L)	4.9 ± 2.4	3.6 ± 1.8	3.8 ± 2.2	0.103		0.408	
	Lym (10^9^/L)	1.2 ± 0.7	1.6 ± 0.7	1.5 ± 0.7	0.265		0.211	
	Mon (10^9^/L)	0.5 ± 0.2	0.4 ± 0.2	0.4 ± 0.2	**0.017**	0.136	0.828	
	TBIL (μmol/L)	8.6 (6.1–13.6)	8.3 (4.8–15.9)	11.1 (6.2–16.3)	0.442		**0.045**	0.360
	DBIL (μmol/L)	3.8 (3.3–8.3)	4.3 (2.8–6.9)	4.9 (3.2–8.7)	0.505		0.048	
	ALT (U/L)	155.0 (144.0–268.0)	146.5 (114.8–219.5)	156.0 (122.0–243.0)	0.664		0.286	
	AST (U/L)	87.0 (76.0–142.0)	96.0 (56.0–215.5)	98.0 (66.0–191.0)	0.575		0.461	
	GGT (U/L)	44.0 (21.0–74.0)	56.0 (27.0–111.0)	59.0 (32.0–97.0)	0.313		0.421	
	ALP (U/L)	65.0 (14.0–112.0)	73.0 (26.0–93.0)	82.0 (55.0–118.8)	0.253		**0.004**	**0.032**
	Total protein (g/L)	62.6 ± 20.4	66.9 ± 17.7	63.0 ± 21.9	0.959		0.079	
	Albumin (g/L)	37.8 ± 7.8	39.3 ± 11.7	38.7 ± 12.4	0.801		0.664	
	Globulin (g/L)	27.4 ± 15.4	26.6 ± 8.8	25.1 ± 10.3	0.466		0.161	

*Note:* Normally distributed variables are presented as mean ± standard deviations (SD), and non‐normally distributed variables as median (interquartile range, IQR). Bold values indicate statistical significance p < 0.05.

Abbreviations: ALP, alkaline phosphatase; ALT, alanine aminotransferase; AST, aspartate aminotransferase; ATDILI, anti‐tuberculosis drug‐induced liver injury; CRP, C‐reactive protein; DBIL, direct bilirubin; ESR, erythrocyte sedimentation rate; GGT, gamma‐glutamyl transferase; Lym, lymphocytes; Mon, monocytes; NA, not available; Neu, neutrophils; SNP, single nucleotide polymorphism; TBIL, total bilirubin; WBC, white blood cell.

^a^ *p* value for the comparison between the mm (homozygous minor allele) genotype and the MM (homozygous major allele) genotype.

^b^ *p* value for the comparison between the mM (heterozygous) genotype and the MM genotype. Multiple comparisons were adjusted using the Bonferroni correction.

In *NAT2* gene polymorphisms, most ATDILI patients with the rs1799931 AA genotype suffered from lung cavitation (75.0%, 6/8), which was a considerably higher rate than that in rs1799931 GG genotype carriers (25.0%, 69/276), with a *p* value of 0.005 (Bonferroni‐adjusted *p* of 0.040, Supporting Information S1: Table S6). In addition, ATDILI cases carrying the rs1799931 AA genotype showed significantly lower levels of total protein (TP) and albumin (ALB) than those with the GG genotype (*p* = 0.002, *p* < 0.001, respectively, Figure 2 and Table 5). Moreover, significantly lower serum TP and ALB levels were also found in ATDILI patients with the rs1799930 AA genotype compared to those with the rs1799930 GG genotype (*p* = 0.032, *p* = 0.004, respectively, Figure 2 and Table 5).

We conducted a detailed investigation to understand how *HLA‐DOA* gene variations relate to the clinical characteristics of ATDILI patients, as illustrated in Table 6, Supporting Information S1: Table S7, and Figure 3. Concerning the ATDILI group, we observed that patients with varying genotypes of the *HLA‐DOA* gene exhibited notable differences in serum TBIL, direct bilirubin (DBIL), and ALP levels. Specifically, ATDILI patients with the TC genotype of rs1367731 showed lower levels of TBIL, DBIL, and ALP compared to those with the CC genotype (*p* = 0.033, *p* = 0.035, *p* = 0.039, respectively). Additionally, significantly lower serum ALP concentrations were found in ATDILI patients with the rs376892 AA genotype compared to those with the GG genotype (*p* = 0.022). The ALP concentrations exhibited a gradual increase from the TT (homozygous minor allele) to TC (heterozygotes), and CC (major homozygous allele) genotypes in SNPs rs6913008 and rs9276975 (Table 6 and Figure 2). Notably, individuals with the rs9276975 TT genotype showed significantly lower serum TBIL and DBIL levels (*p* = 0.031 and 0.009, respectively) in ATDILI. With respect to rs9276977, individuals with heterozygous GA genotypes exhibited significantly reduced levels of ALP and TBIL compared to those with homozygous major GG genotypes (*p* = 0.004, *p* = 0.045, respectively); significantly elevated white blood cell (WBC) and monocytes (Mon) counts were observed in the peripheral circulation of the homozygous AA genotype carriers (*p* = 0.029, *p* = 0.017, respectively). Unfortunately, these significant associations disappeared after the correction for multiple testing.

### 3.4 Functional Annotation of *HLA‐DOA* Genetic Variants

We found potential associations between the *HLA‐DOA* loci rs1367731, rs6913008, and rs9276975 and ATDILI risk in this analysis above. The eQTL analyses revealed that the rs1367731, rs6913008, and rs9276975 variants were eQTLs for *HLA‐DOA* in pancreas tissue and for *HLA‐DPA1* in spleen tissue, as displayed in Supporting Information S1: Table S8A. The allele‐specific microRNA‐binding predictions for SNPs were obtained from the SNPinfo web server database, displaying that rs376892, rs9276975, and rs9276977 exhibited putative microRNA binding sites (Supporting Information S1: Table S8B). Some *HLA‐DQA1/DQB1* alleles were significantly associated with ATDILI (Supporting Information S1: Table S9). We looked into the possible interference effects between *HLA‐DOA* SNPs included in our study and previously reported *HLA‐DQA1/DQB1* variants. However, as shown in Supporting Information S1: Figure S1, those reported *HLA‐DQA1/DQB1* variants were in a long genetic distance and were not in LD with the *HLA‐DOA* SNPs analyzed.

## 4 Discussion

Worldwide, DILI is one of the most common and significant causes of acute liver failure and acute hepatitis. The incidence of DILI is approximately 23.8/100,000 in China and is still rising, which is higher than in Western countries; anti‐TB drugs are the second leading cause of drug‐induced hepatotoxicity in China and account for 21.99% of total DILI [6]. In the current investigation, the occurrence of ATDILI (28.4%) was found to be consistent with previously documented literature (2%–36%) due to the different definitions used, various drug regimens involved, heterogeneous ethnicity, and different sample sizes. Male gender, previous TB treatment, low‐grade fever, hemoptysis, and elevated baseline ALT were found to be independent risk factors for the occurrence of ATDILI in the present study, and the results were comparable to other studies [24]. Notably, a higher ATDILI occurrence rate was observed in males than in females, which may be attributed to the higher TB incidence in male populations. Patients at greater risk of developing ATDILI should receive more attention; more vigilant monitoring is recommended for these patients.

Acetylhydrazine is widely recognized as a primary isoniazid metabolite that contributes to drug‐caused liver injury in anti‐TB therapy. This metabolite is generated by NAT2 and is subjected to further metabolism by cytochrome P450 to form a toxic metabolite or by NAT2 to form the less toxic diacetylhydrazine [25]. It has been proposed that individuals with normal NAT2 activity, that is, fast acetylators, are capable of efficiently producing the less toxic diacetylhydrazine, while those with deficient NAT2 activity, that is, slow acetylators, may produce higher concentrations of the toxic metabolite hydrazine. Recently, a GWAS involving ATDILI patients from Thailand reported a genome‐wide significant signal for *NAT2* slow acetylator phenotypes, demonstrating the significant role of the *NAT2* gene in the etiology of ATDILI [12]. The present study determined that the rs1799930 AA genotype was a potential pharmacogenomic predictor for developing ATDILI (OR = 2.19, 95% CI: 1.40–3.45), which was consistent with Zhang et al.'s findings [26]. Additionally, ATDILI cases showed enriched *NAT2* slow acetylators with an OR of 1.84, which was inferred from the 4‐SNP panel genotypic definition of slow acetylation.

*NAT2* gene polymorphisms have been reported to be associated with clinical variables of ATDILI [27]. In this study, we identified an association between *NAT2* gene variants and adverse clinical phenotypes. Specifically, ATDILI patients carrying the homozygous genotypes rs1799931 AA and rs1799930 AA exhibited significantly decreased serum levels of TP and ALB. Devarbhavi et al. found significantly lower TP and ALB levels in non‐survivors among DILI patients in India [28]. Recently, they further reported that lower serum protein and albumin were significantly associated with mortality in drug‐induced acute liver failure [29]. Moreover, ATDILI patients with the rs1799931 AA genotype had a significantly higher rate of lung cavitation (75.0%) compared to rs1799931 GG genotype carriers (25.0%). Lung cavitation damage is significantly associated with TB treatment failure, indicating a poor prognosis in patients suffered from TB [30]. Furthermore, the rs1799931 AA and rs1799930 AA genotypes have been reported to be negative prognostic factors in colorectal cancer [31]. Considering the above findings, *NAT2* genetic variants were speculated to not only influence the risk of ATDILI development but may also be correlated with poor clinical manifestations and outcomes for ATDILI. However, these results are derived from a small sample‐size observational cohort; our findings should be verified in future studies.

The *HLA‐DOA* on chromosome 6p21.32, a non‐classical *HLA* gene, plays a crucial role as a modulator in the HLA class II‐restricted antigen presentation pathway. HLA‐DO (consisting of HLA‐DOA and HLA‐DOB) both constrains and skews the HLA class II‐presented antigenic peptide repertoire in B cells [32]. Additionally, HLA‐DO may have suppressive effects on autoimmunity [20]. Yao et al. reported that the *HLA‐DOA* rs1044429 AA genotype is correlated with a sustained virological response to HCV treatment with pegylated interferon‐alpha and ribavirin in the Chinese Han population [33]. Sun et al. showed that women with coronary artery disease who have the *HLA‐DOA* rs13210472 C allele are at higher risk for cancer when taking statins [34]. For the first time, our study found a potential link between reduced ATDILI risk and the *HLA‐DOA* genetic markers in the Chinese Han cohort; notably, all three markers (rs1367731, rs6913008, and rs9276975) showed an association in a recessive genotypic model. The recessive model indicated that patients carrying the favorable genotypes at rs1367731 TT, rs6913008 TT, and rs9276975 TT were less susceptible to developing ATDILI (OR = 0.41, 95% CI: 0.18–0.93; OR = 0.39, 95% CI: 0.17–0.89; OR = 0.47, 95% CI: 0.23–0.98, respectively).

The rs1367731 (C > T) is an intergenic variant between *HLA‐DOA* and *HLA‐DPA1*. *HLA‐DOA* rs6913008 (C > T) is located in the intron region, while rs9276975 (C > T) is located at the 3′ UTR. Previous significant *HLA‐DQA1/DQB1* alleles that have been reported to be directly associated with ATDILI are not in LD with *HLA‐DOA* SNPs rs1367731, rs6913008, and rs9276975. The eQTL analyses showed that rs1367731, rs6913008, and rs9276975 are the potential eQTL loci of *HLA‐DOA* and *HLA‐DPA1*, which can affect the gene expression of *HLA‐DOA* and *HLA‐DPA1* in human pancreas and spleen tissues (Table 7B). Both *HLA‐DOA* and *HLA‐DPA1* are located within the MHC class II region, which is involved in immune and inflammation responses [35]. *HLA‐DOA* genetic variations may modulate the expression of MHC class II genes, thereby influencing the activation of immune and inflammatory responses, and contributing to the pathogenesis of ATDILI. Our results preliminarily suggest a potential functional mechanism for the involvement of *HLA‐DOA* variants in ATDILI etiology, and further study is urgently required to confirm this hypothesis. Reynolds et al. found that the rs9276977 minor allele may be significantly associated with rheumatoid arthritis [36]. Furthermore, significantly higher peripheral WBC and Mon counts were found in ATDILI patients who possessed the rs9276977 AA genotype compared to those with the rs9276977 GG genotype, further providing supporting evidence that *HLA‐DOA* variants might regulate immune and inflammation responses that are involved in the development of ATDILI.

Studies have shown that genetic differences in patients can affect how they respond to anti‐TB medications and their risk of adverse liver damage [37]. In this study, ATDILI patients with different genotypes of *HLA‐DOA* variants showed notable differences in serum TBIL, DBIL, and ALP levels. ATDILI cases with the rs1367731 TC genotype and the rs9276977 GA genotype exhibited a marked decrease in ALP and TBIL concentrations. Whereas gradually lower ALP concentrations were observed across the CC, TC, and TT genotypes of rs6913008, commensurate with the rs9276975 analysis. These findings potentially indicated that the *HLA‐DOA* minor alleles could serve as possible factors associated with milder ATDILI disease. These results provided additional preliminary evidence to support our potentially significant association testing results, which suggested that *HLA‐DOA* variants may be possible protective factors and could affect the related clinical manifestations of ATDILI.

Nevertheless, the limitations of the current study should be acknowledged. First of all, this is a single‐center observational cohort study. Due to incomplete medical records, we were unable to obtain detailed information on the specific regimens and duration of previous anti‐TB treatment, which limited our ability to further stratify the analysis by prior treatment history. Secondly, the specific mechanisms through which these genes influence the clinical manifestations of ATDILI were not thoroughly investigated. Additionally, as our study employed a candidate gene approach, there is a potential for false‐positive findings. Although the identified associations appear biologically reasonable, they require confirmation in studies with larger cohorts. Furthermore, as all patients uniformly received a fixed‐dose combination regimen, we cannot isolate each drug's hepatotoxic effect. The lack of complete post‐injury drug modification records precluded RUCAM assessment and limits our conclusions to association rather than causation. It is worth noting that the strict exclusion criteria were used during study subject enrollment; therefore, future validation studies with broader TB populations (including TB/HIV co‐infections and cases with pre‐treatment liver abnormalities) are needed and would be more clinically relevant and valuable. Most significantly, the functional annotation of HLA‐DOA genetic variants is based on computational predictions rather than direct experimental evidence, highlighting the urgent need for comprehensive functional validation in future research.

## 5 Conclusions

In this study, the incidence of ATDILI was relatively high, and may negatively resulting in poor therapeutic outcome. *NAT2* rs1799930 was identified as a genetic risk factor, while *HLA‐DOA* rs1367731, rs6913008, and rs9276975 were found to be potentially genetic protective factors against the development of ATDILI in the Chinese Han population. *NAT2* gene variants rs1799930 and rs1799931 have been linked to poor clinical presentations, and *HLA‐DOA* gene variants are possibly associated with milder clinical manifestations during ATDILI. This study may help to prevent ATDILI development and improve treatment outcomes. Future studies should focus on deeper functional characterization of the *HLA‐DOA* variants to elucidate the mechanisms underlying ATDILI pathogenesis.

## Author Contributions

B.Y. conceptualized and designed this study. Z.Z., D.W., Y.S., H.R., and Y.Z. acquired, analyzed, and interpreted the data. Z.Z., D.W., and Y.S. drafted this manuscript. Z.Z., Y.S., and H.C. provided critical revisions for the manuscript for important intellectual content. Z.Z. and D.W. completed the statistical analysis. All authors have read and approved the final manuscript.

## Ethics Statement

This study protocol was approved by the Ethics Committee of West China Hospital, Sichuan University (reference no. 829, 2019) and the Ethics Committee of the Public Health Clinical Center of Chengdu (reference no. YJ‐K2022‐06‐01).

## Conflicts of Interest

The authors declare no conflicts of interest.

## Supporting information


**Figure S1:** Long genetic distance between *HLA‐DOA* gene and *HLA‐DQA1/DQB1* genes.**Table S1:** Parameters and their laboratory reference range included in this study. **Table S2:** Independent risk factors (at baseline) associated with ATDILI by multivariate logistic regression analysis. **Table S3:** The information of candidate SNPs. **Table S4:** *NAT2* acetylator phenotypes associated with ATDILI. 4A Distribution of inferred *NAT2* acetylator phenotypes. 4B Inferred *NAT2* acetylator phenotype significantly associated with ATDILI risk. **Table S5:** Haplotype analysis of *HLA‐DOA* gene in ATDILI patients and non‐ATDILI controls. **Table S6:** Analysis of the association between *NAT2* SNPs and clinical features in ATDILI patients. **Table S7:** Analysis of the association between *HLA‐DOA* SNPs and clinical features in ATDILI patients. **Table S8:** Functional annotation of *HLA‐DOA* genetic markers. S8A Analyses of eQTL in *HLA‐DOA* markers potentially associated with ATDILI risk. S8B The functional prediction of rs376892, rs9276975, and rs9276977 from SNPinfo web server. **Table S9:** Annotations for candidate *HLA* genetic variants of DILI

Supporting File

## Data Availability

The data that support the findings of this study are available from the corresponding author upon reasonable request.
