# Supplementary material for: Genetic Variants at the NAT2 and HLA‐DOA are Associated With Anti‐Tuberculosis Drug‐Induced Liver Injury Susceptibility and Clinical Manifestations in Western Chinese Populations
Source: Immun Inflamm Dis. 2026 Sep 15;14(9):e70517. doi: 10.1002/iid3.70517 (PMC13576906; doi:10.1002/iid3.70517)
Supplement: Supplementary file 2 [file IID3-14-e70517-s002.docx]

**Genetic Variants at the *NAT2* and *HLA-DOA* are associated with ATDILI susceptibility and clinical manifestations in Western Chinese Populations**

**Supplementary Table S1** Parameters and their laboratory reference range included in this study.

| Parameters | Abbreviations | Unit | Normal range |
| --- | --- | --- | --- |
| white blood cell counts | WBC | ×10^9^/L | 3.50-9.50 |
| neutrophils | Neu | ×10^9^/L | 1.80-6.30 |
| lymphocytes | Lym | ×10^9^/L | 1.10-3.20 |
| monocytes | Mon | ×10^9^/L | 0.10-0.60 |
| eosinophils | Eos | ×10^9^/L | 0.02-0.52 |
| red blood cell count | RBC | ×10^12^/L | 4.30-5.80 |
| hemoglobin | Hb | g/L | 130-175 |
| hematocrit | Hct | % | 40-50 |
| platelet | PLT | ×10^9^/L | 125-350 |
| total bilirubin | TBIL | μmol/L | 0-20.5 |
| direct bilirubin | DBIL | μmol/L | 0-6.8 |
| indirect bilirubin | IBIL | μmol/L | 0-20 |
| alanine aminotransferase | ALT | U/L | 0-37 |
| aspartate aminotransferase | AST | U/L | 0-37 |
| AST/ALT |  |  | 0.8-1.5 |
| gamma-glutamyl transpeptidase | GGT | U/L | 0-50 |
| alkaline phosphatase | ALP | U/L | 40-150 |
| total protein | TP | g/L | 60-85 |
| albumin | ALB | g/L | 35-55 |
| globulin | GLB | g/L | 20-35 |
| ALB/GLB |  |  | 1.1-2.5 |
| serum calcium | CA | mmol/L | 2.20-2.55 |
| glucose | GLU | mmol/L | 3.9-6.1 |
| urea | UREA | mmol/L | 2-6.9 |
| creatinine | CREA | μmol/L | 40-133 |
| cystatin C | Cys-c | mg/L | 0.51-1.09 |
| prothrombin time | PT | s | 11-16 |
| international normalized ratio | INR |  | 0.8-1.3 |
| activated partial thromboplastin time | APTT | s | 27-45 |
| thrombin time | TT | s | 10-20 |
| c-reactive protein | CRP | mg/L | 0-6 |
| erythrocyte sedimentation rate | ESR | mm/h | <15 |

**Supplementary Table S2** Independent risk factors (at baseline) associated with ATDILI by multivariate logistic regression analysis.

| Characteristic | OR | 95% CI | *P* value |
| --- | --- | --- | --- |
| Male | 2.35 | 1.77-3.11 | **<0.001** |
| Previous TB treatment | 2.53 | 1.78-3.60 | **<0.001** |
| Low-grade fever | 1.52 | 1.12-2.07 | **0.007** |
| Hemoptysis | 1.55 | 1.05-2.29 | **0.029** |
| Baselin ALT≧18.4(U/L) | 2.30 | 1.69-3.12 | **<0.001** |

Note: Abbreviations: ATDILI: anti-tuberculosis drug-induced liver injury; TB: tuberculosis; ALT: alanine aminotransferase.

**Supplementary Table S3** The information of candidate SNPs.

| SNP | Chr: position | Stand | Functional Consequence | Amino acid change |
| --- | --- | --- | --- | --- |
| *NAT2* gene |  |  |  |  |
| rs1801280 T > C | 8:18400344 | + | missense variant | I/T |
| rs1799930 G > A | 8:18400593 | + | missense variant | R/Q |
| rs1799931 G > A | 8:18400860 | + | missense variant | G/E |
| rs1801279 G > A | 8:18400194 | + | missense variant | R/Q |
| *HLA-DOA* gene |  |  |  |  |
| rs1367731 C > T | 6:33017422 | + | intergenic region variant（*HLA-DOA*-*HLA-DPA1*） | - |
| rs376892 G > A | 6:33005110 | + | 3 prime UTR variant | - |
| rs6913008 C > T | 6:33007066 | + | intron variant | - |
| rs9276975 C > T | 6:33005822 | + | 3 prime UTR variant | - |
| rs9276977 G > A | 6:33006064 | + | 3 prime UTR variant | - |

Note: Abbreviations: SNP: single nucleotide polymorphism; Chr: chromosome; UTR: untranslated region; I: isoleucine; T: threonine; R: arginine; Q: glutamine; G: glycine; E: glutamic acid.

**Supplementary Table S4** *NAT2* acetylator phenotypes associated with ATDILI.

**4A** Distribution of inferred *NAT2* acetylator phenotypes.

| Acetylator status | Overall(n=1358) | ATDILI(n=385) | non-ATDILI (n=973) |
| --- | --- | --- | --- |
| Rapid acetylator (RA) | 402(29.6%） | 108(28.1%) | 294(30.2%) |
| Intermediate acetylator (IA) | 692(51.0%) | 174(45.2%) | 518(53.2%) |
| Slow acetylator (SA) | 264(19.4%) | 103(26.7%) | 161(16.6%) |

**4B** Inferred *NAT2* acetylator phenotype significantly associated with ATDILI risk.

| Acetylator status | ATDILI(n=385) | non-ATDILI (n=973) | *P* value | OR (95% CI) |
| --- | --- | --- | --- | --- |
| Slow acetylator (SA) | 103(26.8%) | 161(16.5%) | **<0.001** | 1.84(1.39-2.44) |
| Intermediate &  rapid acetylator  (IA & RA) | 282(73.2%) | 812(83.5%) |  |  |

**Supplementary Table S5** Haplotype analysis of *HLA-DOA* gene in ATDILI patients and non-ATDILI controls.

| Haplotype | ATDILI cases(n%) | non-ATDILI controls(n%) | *P* value |
| --- | --- | --- | --- |
| rs9276975-rs6913008-1367731 |  |  |  |
| CCC | 615(82.0) | 1514(77.8) | 0.256 |
| TTT | 139(18.5) | 391(20.1) | 0.222 |
| CCT | 12(1.6) | 30(1.5) | 0.977 |

**Supplementary Table S6** Analysis of the association between *NAT2* SNPs and clinical features in ATDILI patients.

| SNP | Clinical indices | ATDILI patients (n=385) | | | *P* value ^a^ | Bonferroni | *P* value ^b^ |
| --- | --- | --- | --- | --- | --- | --- | --- |
|  |  | Genotype | | |  | *P* value ^a^ |  |
| rs1801280 |  | CC(n=1) | TC(n=36) | TT(n=348) |  |  |  |
|  | Cough | 1(100.0) | 25(69.4) | 242(69.5) | NA |  | 0.991 |
|  | Low-grade Fever | 0 | 12(33.3) | 100(28.7) | NA |  | 0.563 |
|  | Lung Cavitation | 0 | 7(19.4) | 94(27.0) | NA |  | 0.326 |
|  | Pleural effusion | 0 | 9(25.0) | 99(28.4) | NA |  | 0.661 |
|  | Liver injury pattern |  |  |  | NA |  | 0.415 |
|  | Hepatocellular | 0 | 19(52.8) | 219(62.9) |  |  |  |
|  | Cholestatic | 0 | 1(2.8) | 18(5.2) |  |  |  |
|  | Mixed | 1(100.0) | 13(36.1) | 96(27.6) |  |  |  |
| rs1799930 |  | AA(n=38) | GA(n=149) | GG(n=198) |  |  |  |
|  | Cough | 27(71.1) | 103(69.1) | 138(69.7) | 0.867 |  | 0.909 |
|  | Low-grade Fever | 12(31.6) | 40(26.8) | 60(30.3) | 0.876 |  | 0.482 |
|  | Lung Cavitation | 8(21.1) | 43(28.9) | 50(25.3) | 0.582 |  | 0.453 |
|  | Pleural effusion | 15(39.5) | 40(26.8) | 53(26.8) | 0.113 |  | 0.987 |
|  | Liver injury pattern |  |  |  | 0.498 |  | 0.578 |
|  | Hepatocellular | 24(63.2) | 96(64.4) | 118(59.6) |  |  |  |
|  | Cholestatic | 3(7.9) | 7(4.7) | 9(4.5) |  |  |  |
|  | Mixed | 9(23.7) | 39(26.2) | 62(31.3) |  |  |  |
| rs1799931 |  | AA(n=8) | GA(n=101) | GG(n=276) |  |  |  |
|  | Cough | 5(62.5) | 72(71.3) | 191(69.2) | 0.706 |  | 0.696 |
|  | Low-grade Fever | 4(50.0) | 27(26.7) | 81(29.3) | 0.246 |  | 0.619 |
|  | Lung Cavitation | 6(75.0) | 26(25.7) | 69(25.0) | **0.005** | **0.040** | 0.883 |
|  | Pleural effusion | 3(37.5) | 26(25.7) | 79(28.6) | 0.694 |  | 0.581 |
|  | Liver injury pattern |  |  |  | NA |  | 0.581 |
|  | Hepatocellular | 5(62.5) | 61(60.4) | 172(62.3) |  |  |  |
|  | Cholestatic | 0 | 2(2.0) | 17(6.2) |  |  |  |
|  | Mixed | 2(25.0) | 31(30.7) | 77(27.9) |  |  |  |

Note: ^a^ *P* value was the comparison between the mm (homozygous minor allele) genotype and the MM (major homozygous allele) genotype. ^b^ *P* value depicted the comparison between the mM (heterozygotes) genotype and the MM genotype.

**Supplementary Table S7** Analysis of the association between *HLA-DOA* SNPs and clinical features in ATDILI patients.

| SNP | Clinical indices | ATDILI patients (n=385) | | | *P* value ^a^ | *P* value ^b^ |
| --- | --- | --- | --- | --- | --- | --- |
|  |  | Genotype | | |  |  |
| rs1367731 |  | TT(n=7) | TC(n=128) | CC(n=250) |  |  |
|  | Cough | 5(71.4) | 92(71.9) | 171(68.4) | >0.999 | 0.487 |
|  | Low-grade Fever | 2(28.6) | 37(28.9) | 73(28.2) | >0.999 | 0.953 |
|  | Lung Cavitation | 1(14.3) | 30(23.4) | 70(28.0) | 0.677 | 0.341 |
|  | Pleural effusion | 3(42.9) | 32(25.0) | 73(29.2) | 0.425 | 0.388 |
|  | Liver injury pattern |  |  |  | NA | 0.957 |
|  | Hepatocellular | 5(71.4) | 79(61.7) | 154(61.6) |  |  |
|  | Cholestatic | 0 | 7(5.5) | 12(4.8) |  |  |
|  | Mixed | 2(28.6) | 36(28.1) | 72(28.8) |  |  |
| rs376892 |  | AA(n=20) | GA(n=149) | GG(n=216) |  |  |
|  | Cough | 12(60.0) | 110(73.8) | 146(67.6) | 0.490 | 0.201 |
|  | Low-grade Fever | 6(30.0) | 43(28.9) | 63(29.2) | 0.938 | 0.949 |
|  | Lung Cavitation | 5(25.0) | 33(22.1) | 63(29.2) | 0.694 | 0.134 |
|  | Pleural effusion | 3(15.0) | 41(27.5) | 64(29.6) | 0.165 | 0.661 |
|  | Liver injury pattern |  |  |  | 0.578 | 0.581 |
|  | Hepatocellular | 13(65.0) | 95(63.8) | 130(60.2) |  |  |
|  | Cholestatic | 2(10.0) | 7(4.7) | 10(4.6) |  |  |
|  | Mixed | 5(25.0) | 38(25.5) | 67(31.0) |  |  |
| rs6913008 |  | TT(n=7) | TC(n=128) | CC(n=250) |  |  |
|  | Cough | 5(71.4) | 92(71.9) | 171(68.4) | >0.999 | 0.487 |
|  | Low-grade Fever | 2(28.6) | 37(28.9) | 73(29.2) | >0.999 | 0.953 |
|  | Lung Cavitation | 1(14.3) | 31(24.2) | 69(27.6) | 0.678 | 0.481 |
|  | Pleural effusion | 3(42.9) | 32(25.0) | 73(29.2) | 0.425 | 0.388 |
|  | Liver injury pattern |  |  |  | NA | 0.905 |
|  | Hepatocellular | 5(71.4) | 80(62.5) | 153(61.2) |  |  |
|  | Cholestatic | 0 | 7(5.5) | 12(4.8) |  |  |
|  | Mixed | 2(28.6) | 35(27.3) | 73(29.2) |  |  |
| rs9276975 |  | TT(n=9) | TC(n=134) | CC(n=242) |  |  |
|  | Cough | 7(77.8) | 94(70.1) | 167(69.0) | 0.726 | 0.818 |
|  | Low-grade Fever | 2(22.2) | 38(28.4) | 72(29.8) | >0.999 | 0.776 |
|  | Lung Cavitation | 1(11.1) | 30(22.4) | 70(28.9) | 0.452 | 0.169 |
|  | Pleural effusion | 3(33.3) | 34(25.4) | 71(29.3) | 0.726 | 0.412 |
|  | Liver injury pattern |  |  |  | NA | 0.641 |
|  | Hepatocellular | 7(77.8) | 84(62.7) | 147(60.7) |  |  |
|  | Cholestatic | 0 | 8(6.0) | 11(4.5) |  |  |
|  | Mixed | 2(22.2) | 35(26.1) | 73(30.2) |  |  |
| rs9276977 |  | AA(n=11) | GA(n=134) | GG(n=240) |  |  |
|  | Cough | 8(72.7) | 97(72.4) | 163(67.9) | >0.999 | 0.368 |
|  | Low-grade Fever | 3(27.3) | 41(30.6) | 68(28.3) | >0.999 | 0.644 |
|  | Lung Cavitation | 3(27.3) | 32(23.9) | 66(27.5) | >0.999 | 0.445 |
|  | Pleural effusion | 4(36.4) | 33(24.6) | 71(29.6) | 0.737 | 0.305 |
|  | Liver injury pattern |  |  |  | 0.865 | 0.793 |
|  | Hepatocellular | 7(63.6) | 86(64.2) | 145(60.4) |  |  |
|  | Cholestatic | 1(9.1) | 6(4.5) | 12(5.0) |  |  |
|  | Mixed | 3(27.3) | 36(26.9) | 71(29.6) |  |  |

Note: ^a^ *P* value was the comparison between the mm (homozygous minor allele) genotype and the MM (major homozygous allele) genotype. ^b^ *P* value depicted the comparison between the mM (heterozygotes) genotype and the MM genotype.

**Supplementary Table S8** Functional annotation of *HLA-DOA* genetic markers.

**8A** Analyses of eQTL in *HLA-DOA* markers potentially associated with ATDILI risk.

| SNP | Gene Symbol | *P*-Value | Effect Size | Tissue |
| --- | --- | --- | --- | --- |
| rs1367731 | *HLA-DPA1* | 4.20E-06 | 0.28 | Spleen |
|  | *HLA-DOA* | 6.30E-06 | -0.34 | Pancreas |
| rs6913008 | *HLA-DPA1* | 1.90E-06 | 0.29 | Spleen |
|  | *HLA-DOA* | 3.80E-06 | -0.35 | Pancreas |
| rs9276975 | *HLA-DPA1* | 2.80E-06 | 0.29 | Spleen |
|  | *HLA-DOA* | 1.80E-05 | -0.33 | Pancreas |

**8B** The functional prediction of rs376892, rs9276975, and rs9276977 from SNPinfo web server.

| SNP | Allele | Position | Prediction Strand | Forward Sequence | microRNA | Score | Energy |
| --- | --- | --- | --- | --- | --- | --- | --- |
| rs376892 | A | 7 | - | aCCCCCAATGGCACCTGATTa | miR-361-3p | 146 | -24.31 |
| rs9276975 | C | 3 | - | gtCCTGTCCCCTTGGCCTCCCA | miR-339-5p | 141 | -24.4 |
| rs9276977 | G | 5 | - | TAGAGTGTGTCTAGGTGgacc | miR-122 | 149 | -19.88 |
| rs9276977 | A | 5 | - | tAGAATGTGTCTAGGTGgacct | miR-1265 | 141 | -12.8 |
| rs9276977 | A | 14 | - | CCACATATTTAGAATGTgtcta | miR-448 | 142 | -14.36 |
| rs9276977 | A | 18 | - | taGGCCACATATTTAGAAt | miR-588 | 140 | -18.37 |
| rs9276977 | G | 4 | - | agAGTGTGTCTAGGTGGACCT | miR-595 | 145 | -16.91 |

**Supplementary Table S9** Annotations for candidate *HLA* genetic variants of DILI.

| **Gene** | **Variant** | **Chromosome** | **dbSNP** | **Drugs** |
| --- | --- | --- | --- | --- |
|  |  |  | **function** |  |
| *HLA-DQA1* | rs9270986(A>C) | 6 | intergenic variant | anti-TB drugs |
| *HLA-DQB1* | rs1063355(T>G) | 6 | 3 prime UTR variant | anti-TB drugs |
| *LTA* | rs1041981(C>A) | 6 | missense variant | anti-TB drugs |
| *HLA-B* | *HLA-B*52:01* | 6 | variation | anti-TB drugs |
| *HLA-C* | *HLA-C*12:02* | 6 | variation | anti-TB drugs |
| *HLA-DQA1* | *HLA-DQA1*01:02* | 6 | variation | anti-TB drugs |
| *HLA-DQB1* | *HLA-DQB1*02:01* | 6 | variation | anti-TB drugs |
| *HLA-DQB1* | *HLA-DQB1*05:02* | 6 | variation | anti-TB drugs |
| *HLA-B* | *HLA-B*08:01* | 6 | variation | infliximab |
| *HLA-B* | *HLA-B*39:01* | 6 | variation | infliximab |
| *HLA-B* | *HLA-B*35:02* | 6 | variation | minocycline |
| *HLA-B* | *HLA-B*57:01* | 6 | variation | flucloxacillin |
| *HLA-C* | *HLA-C*12:03* | 6 | variation | infliximab |
| *HLA-DQB1* | *HLA-DQB1*05:02* | 6 | variation | flupirtine |
| *HLA-DQB1* | *HLA-DQB1*02:01* | 6 | variation | infliximab |
| *HLA-DRB1* | *HLA-DRB1*03:01* | 6 | variation | infliximab |
| *HLA-DRB1* | *HLA-DRB1*04:04* | 6 | variation | infliximab |
| *HLA-DRB1* | *HLA-DRB1*16:01* | 6 | variation | flupirtine |

Note: We selected the *HLA* alleles which were well studied and found directly associated with DILI, by searching for the pharmaGKB database, the dbSNP database and the Pubmed database.

**Figure Legends**

Supplementary Figure S1. Long genetic distance between *HLA-DOA* gene and *HLA-DQA1/DQB1* genes.

Supplementary Figure 1.
